# Non-ambulatory children with cerebral palsy: effects of four months of static and dynamic standing exercise on passive range of motion and spasticity in the hip

**DOI:** 10.7717/peerj.8561

**Published:** 2020-03-17

**Authors:** Åsa B. Tornberg, Katarina Lauruschkus

**Affiliations:** Department of Health Sciences, Lund University, Lund, Sweden

**Keywords:** Children, Cerebral palsy, Passive range of motion, Spasticity, Standing frame

## Abstract

**Purpose:**

The aim of this study was to compare the effects of four months of two types of structured training regimes, static standing (StS) versus dynamic standing (DyS), on passive range of motion (PROM) and spasticity in the hip among non-ambulatory children with cerebral palsy.

**Method:**

Twenty non-ambulatory children with cerebral palsy participated in an exercise intervention study with a crossover design. During StS, the Non-ambulatory children with cerebral palsy were encouraged to exercise according to standard care recommendations, including daily supported StS for 30–90 min. During DyS, daily exercise for at least 30 min at a speed between 30 and 50 rpm in an Innowalk (Made for movement, Norway) was recommended. We assessed adaptive effects from the exercise programs through PROM in the hip assessed with a handheld goniometer, and spasticity in the hip assessed with the Modified Ashworth Scale before and after 30 min of StS or DyS. A trained physiotherapist performed the assessments. The exercise test and exercise training were performed in the children’s habitual environment. Non-parametric statistics were used and each leg was used as its own control.

**Result:**

PROM increased in all directions after 30 min (*p* < 0.001), and after four months of exercise training (*p* < 0.001) of DyS. Thirty minutes of DyS lowered the spasticity in the muscles around the hip (*p* < 0.001) more than 30 min of StS (*p* < 0.001).

**Conclusion:**

Thirty minutes of DyS increased PROM and decreased spasticity among non-ambulatory children with CP. Four months of DyS increased PROM but did not decrease spasticity. These results can help inform individualised standing recommendations.

## Introduction

**Figure 1:**
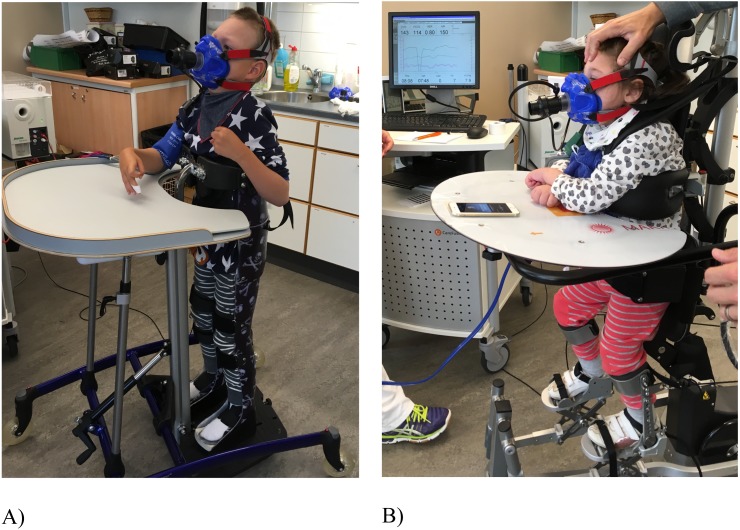
(A) Static standing (StS) in a standing frame and (B) dynamic standing (DyS) in the Innowalk.

Non-ambulatory children with cerebral palsy cannot walk or sit without support, and their gross motor function is classified as level IV and V according to the five level classification system GMFCS-E&R, where level V implies the most severe function limitations (Palisano et al., 2008). Therefore, these children have difficulties being physical active. There are few high-quality exercise intervention studies on children with CP, and those that have been performed have mainly been on ambulatory children with CP (Ryan et al., 2017). Standard care in Sweden for Na-CP includes standing exercise training in standing frames for 45–90 min daily, in accordance with evidence-based recommendations (Paleg, Smith & Glickman, 2013). The standing exercise training in standing frames is a static standing (StS) exercise where the child is fixated in an individually casted frame or in another standing device. No lower body movements can be achieved but standing in an upright position is possible (Fig. 1A). In a feasibility study (Lauruschkus et al., 2017) about how to promote physical activity among children with CP, five children exercised with the motorised medical device Innowalk (Fig. 1B). Innowalk gives an opportunity to experience walking movements in an upright weight-bearing position, making dynamic standing (DyS) possible. The children enjoyed the exercise training and their parents reported improved gastrointestinal function, warmer feet, and improved motor function compared with StS. It seems as if StS and DyS represent two different exercise training modalities for non-ambulatory children with CP, and more knowledge about the two interventions are needed. The physiological effects of training in StS compared to DyS are not known.

Earlier studies on Passive Range Of Motion (PROM) have mainly been on the effects of CP severity and age. McDowell et al. (2012) showed that PROM of abduction, internal rotation and external rotation decreased with higher GMFCSE&R level (McDowell et al., 2012), and Nordmark et al. (2009) showed decrements in abduction and external rotation between the ages of two and fourteen (Nordmark et al., 2009). Fragala, Goodgold & Dumas (2003) performed a multiple case study on exercise showing that exercise improves PROM.

Spasticity has been defined as a velocity-dependent increase in the tonic stretch reflex as a result of hyper excitability of the reflex (Lance, 1980). Even at low velocities, an increase in the activation of the tonic stretch reflex has been seen (Thilmann, Fellows & Garms, 1991; Van den Noort et al., 2010; Bar-On et al., 2013). Additionally, the activation of the reflex continues after the movement has stopped (Sheean, 2002). It has also been reported that the size and number of muscle lengthening are related to the amount of tonic stretch reflex activation (Levin & Feldman, 1994; Malhotra et al., 2008).

To the best of our knowledge, no earlier studies comparing the long-term exercise effects from StS versus DyS on PROM and spasticity in children with CP GMFCS-E&R level and V have been performed.

Therefore, the aim of this study was to elucidate the effects of a four-month exercise regime with StS and a four-month exercise regime with DyS on PROM and spasticity in the hips among non-ambulatory children with CP GMFCS-E&R level IV and V.

## Materials & Methods

### Setting

The assessments before, during, and after the standing interventions and the exercise interventions were performed in the children’s habitual environments, either at home or at their school, which is also where the questionnaires were filled in. This home-setting facilitated the involvement of the children during the study period. KL, a registered pedriactic physiotherapist, performed the PROM and spasticity assessments and supported the families during the intervention. The study was approved by the Regional Ethics Committee in Lund, Sweden (EPN-dnr 2017/67) and is registered at ISRCTN (ISRCTN10569363). The participants provided written informed consent.

### Population

**Figure 2:**
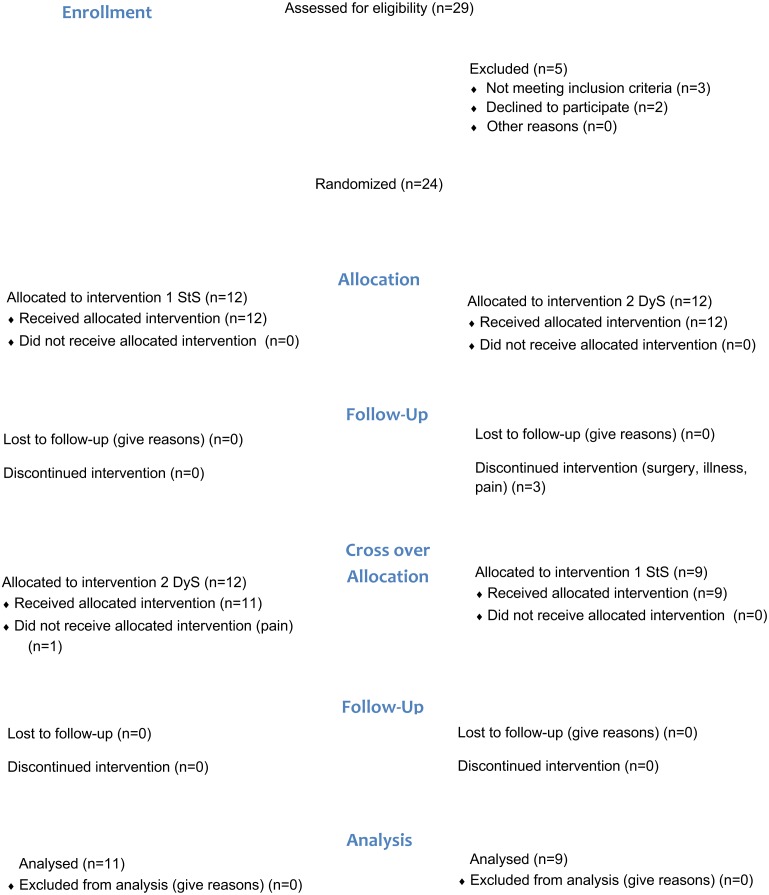
CONSORT flow diagram of the study.

Twenty-four children were initially included in the study, and 20 children aged between 5 and 7 years (mean 11.6 ± 3.6 years; 9 female) with CP GMFCS-E&R level IV and V completed the study. The participants were recruited through the Child and Youth Habilitation Services in the Skane Region, Sweden. Seventeen of the 20 participants used augmentative and alternative communication. Participant characteristics are shown in Table 1 and CONSORT workflow in Fig. 2.

**Table 1:** Characteristics of the participants.

**Children (*N* = 20)**	***n***
CP-subtype	
Spastic unilateral	0
Spastic bilateral	14
Dyskinetic	6
Ataxic	0
GMFCS-E&R level	
IV	11
V	9
Cognitive level^a^	
No mental retardation	2
Mild mental retardation	5
Moderate to profound mental retardation	13

**Notes.**

a WHO’s International Classification of Diseases (ICD): ICD-10 codes for mental retardation; the information was reported by parents.

### Study protocol

**Figure 3:**
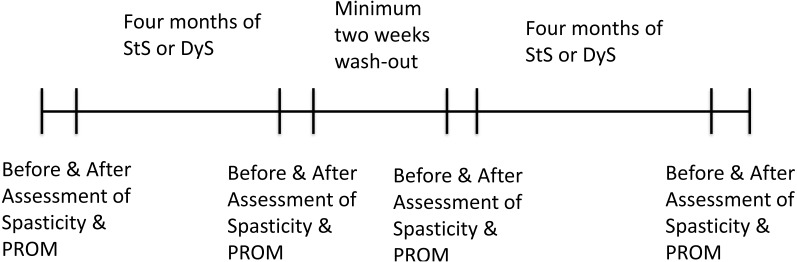
Timeline of the study protocol.

We performed an exercise intervention study with a cross-over design (Fig. 3). Each participant performed four months of either StS or DyS with a wash-out period of a minimum two weeks between the two exercise interventions (Neufer, 1989; Mujika & Padilla, 2000; Coyle et al., 1984). Exercise tests were performed before and after each exercise intervention period.

### Exercise testing

All children were together with their parents and/or their personal assistants, when KL and ÅBT came to their home or school to perform the exercise testing. Assessments of spasticity and PROM were performed before the exercise test. During exercise testing the child was then positioned either in a standing frame or in the motorised medical device Innowalk (Made for Movement, Norway) in a standing position for 30 min. The Innowalk was personally adjusted to each child by a trained technician. During exercise testing, the children were allowed to do their normal activities, as listen to music, play with toys etc. After 30 min of standing the child or adolescent was lifted down on a mat for a second assessment of spasticity and PROM.

### Exercise training

All children had to be assisted during exercise by either a parent, school personnel or a personal assistant. During StS, the instruction was to exercise as usual according to standard care, meaning 30–90 min of static standing per day in a personal casted standing shell or other standing devices. While exercising, the children were allowed to do their normal activities, as listening to music, playing with toys, take part in daily activity in school or at home etc. Before the exercise period with DyS started, the Innowalk was personally adjusted by a skilled technician. During initial two weeks of the DyS period, the instruction was to increase the length of each exercise session, from shorter periods per day until the child had adapted to the DyS. Slower walking speeds, about 30–40 revolutions per minute (rpm) equivalent to 15–20 strides per minute, were also recommended during the first two weeks. After the adaptation period, the instruction was to exercise at least 30, but preferably 45–60 min per day and with a walking speed up to 50 rpm, equivalent to 25 strides per minute. After two months of DyS exercise, KL and a trained technician followed up the exercise and the adjustments of the Innowalk. During exercise, the children were allowed to do their normal activities, as listening to music, playing with toy, take part in daily activity in school or at home etc. The children, through their parents, were also encouraged to walk at different speeds according to their own preferences. Exercise diaries were filled in during each exercise intervention period.

### Assessments

PROM (Fosang et al., 2003) was assessed by the same physiotherapist (KL) at all time points. The child was lying on a flat surface, either on a bed or at the floor on a madras. All children were used to the assessment procedure. PROM was assessed for abduction, flexion, extension, Ely’s test, internal rotation and external rotation in the hip according to the CPUP protocol (www.cpup.se) using a handheld goniometer (before and after exercise testing, before and after four months of StS and DyS exercise training) (Table 2).

Spasticity in hip flexors, extensors and adductors, was assessed before and after StS and DyS, by the Modified Ashworth Scale (Bohannon & Smith, 1987). This scale is as follows:

0 No increase in muscle tone

1 Slight increase in muscle tone, manifests itself as ”catch and release” or as a minimum resistance at the end of the motion path

+1 Slight increase in muscle tone, appears as ”catch” followed by minimal resistance through the rest of the motion path (less than half the range of motion)

2 More marked increase of muscle tone through most of the motion path, but the movement is still easy to perform

3 Significant increase in muscle tone, difficult to perform passive movements

4 Stiffness when trying to flex or extend a body part.

Starting positions used when estimating spasticity were lying in a supine position.

Starting positions used when estimating spasticity were lying in a supine position.

**Table 2:** Child and goniometer positioning and standardization procedure for abduction, flexion, extension, Ely’s test and internal and external rotation.

**Motion of the hip**	**Body and Extremity position**	**Goniometer position: center**	**Goniometer position: stationary arm**	**Goniometer position: movable arm**	**End extremity position**	**Additional standardization**
*Abduction*	Supine. Test leg in natural (extended position)	Anterior superior iliac spines	Along a line joining the two anterior superior iliac spines	Along the femur	Hip abducted to limit of motion	Pelvis stabilized by fixating opposite leg slightly abducted and flexed over edge of plinth.
*Flexion*	Supine. Test leg in natural (extended position)	Trochanter major	Along with the trunk parallel to the columna	Along the femur	Hip flexion to limit of motion	The pelvis was fixated by extending the opposite leg, while the hip and knee of the assessed leg was flexed.
*Extension*	Prone or on the side. Test leg in natural (extended position)	Trochanter major	Along with the trunk parallel to the columna	Along the femur	Hip extension to limit of motion	Pelvis in a flat position. The pelvis was fixated. Any motion limitations up to horizontal mode were indicated by minus.
*Elys test*	Prone. With extended hips and the test leg knee in full extended position.	Knee joint	Along the femur, aiming at trochanter major	Parallel to tibia’s leading edge and aiming towards lateral malleolus	Knee flexion from the straight knee at the level at which the pelvis wants to “lift”	Fixed pelvis while flexing the knee.
*Internal rotation*	Prone. With extended hips and the test leg knee flexed to 90°. Tester holding the tested leg and secure the pelvic rotation by stabilizing the pelvis with the other hand	Knee joint	Parallel to the plinth	Parallel to longitudinal axis of tibia	Internal rotation to limit of motion just before pelvis just starts to lift from plinth	Pelvis in a flat position. The pelvis was fixated.
*External rotation*	Prone. With extended hips and the test leg knee flexed to 90°. Tester holding the tested leg and secure the pelvic rotation by stabilizing the pelvis with the other hand	Knee joint	Parallel to the plinth	Parallel to longitudinal axis of tibia	External rotation to limit of motion just before pelvis just starts to lift from plinth	Pelvis in a flat position. The pelvis was fixated.

*Hip flexors:* the leg was moved in a flexion-extension range of motion to feel the muscle tonus when the hip was extended.

*Hip extensors:* the leg was moved in a flexion-extension range of motion to feel the muscle tonus when the hip was flexed.

*Adductors:* assessed with the child’s hips and knees extended. The leg was moved in an abduction-adduction range of motion, to feel the muscle tone when the leg was abducted.

### Calculations and statistical analysis

Each leg of each child was used as a single event giving in total 40 events at each assessment point.

As data from the exercise dairies of the exercise sessions were considered to be normally distributed, parametric statistics were used. Data for PROM and spasticity were not normally distributed and therefore non-parametric statistics were used.

Descriptive statistics (mean and standard deviation (SD)) were calculated for exercise sessions and median and quartiles for PROM and spasticity.

To compare number of exercise sessions between the two interventions on a group level, a paired Student’s *t*-test was used, since each child was its own control. When analysing the effects on PROM and spasticity, a 2 × 2 repeated measurement Friedman ANOVA was performed and a pairwise Wilcoxon sign test was used as post-hoc test. Analysis of the change in PROM and spasticity before and after an exercise test was performed by pairwise Wilcoxon Signed Ranks Test.

All statistical analyses were performed using SPSS for Windows (version 25.0, SPSS Inc, Chicago, Illinois, USA). Statistical significance was set at *p* < 0.001.

**Table 3:** Shown in the table is number of possible exercise days, days of performed exercise and days of non-exercising. The results from the exercise diaries are shown in the table.

	**Static standing**	**Dynamic standing**	*p*-value
All possible exercise days (N)	117 (13)	120 (20)	0.59
All days exercise performed (N)	63 (32)	83 (27)	0.010
Percent performed exercise of total days (%)	53 (25)	69 (18)	0.008
Total exercise time (Minutes)	3,651 (4,080)	3,734 (2,370)	0.90
Average exercise time (Minutes)	51 (32)	43 (19)	0.10
Maximum exercise time (Minutes)	82 (57)	79 (36)	0.36
Minimum exercise time (Minutes)	24 (14)	14 (6)	0.006
Non-exercising days (N)	55 (29)	38 (21)	0.016
Average non-exercising days (N)	4 (6)	2 (1)	0.083
Maximum non-exercising days (N)	18 (18)	8 (5)	0.019
Minimum non-exercising days	1 (0)	1 (1)	1.0
Number of non-exercising periods (N)	18 (8)	18 (10)	0.83

**Notes.**

All possible exercise days are defined as the total number of days in the exercise period; *All days exercise performed* are the number of day when exercise was performed during the exercise period; *Total exercise time* is the total time of exercise during the exercise period; *Average exercise time* is the average of each exercise session. *Maximum and minimum exercise time* are the max and min of the length of an exercise session; *Non-exercising days* are the number of days when no exercise was performed; *Average non-exercising days* is the average of no exercising days within the group; *Maximum and minimum non-exercising days* are the max and min of no exercising days within the group; *Number of non-exercising periods* is the number of periods with consecutive days of non-exercise. Data are presented as mean and standard deviations (SD). Paired Student *t*-test was used to analyze statistical differences. A *p*-value less than 0.05 was considered statistically significant.

**Table 4:** Assessment of passive range of motion (PROM) in the hip before and after the exercise tests starting and ending the exercise intervention periods of Static (StS) and Dynamic (DyS).

Measure	Exercise intervention	Assessment timepoint before and after the intervention (INT)	Assessment timepoint before and after the exercise test (ET)	Median	Quartiles	
					25th	75th
*Abduction*	StS	Before INT	Before ET	32.5	25	40
			After ET	32.5	30	40
		After INT	Before ET	40	30	40
			After ET	40	30	40
	DyS	Before INT	Before ET	30	25	40
			After ET	40	35	43.75
		After INT	Before ET	35	25	40
			After ET	40	35	45
*Flexion*	StS	Before INT	Before ET	130	120	135
			After ET	130	116.25	135
		After INT	Before ET	130	110	140
			After ET	130	102.5	140
	DyS	Before INT	Before ET	130	120	135
			After ET	135	130	140
		After INT	Before ET	130	112.5	140
			After ET	140	130	140
*Extension*	StS	Before INT	Before ET	0	−5	13.75
			After ET	5	−5	10
		After INT	Before ET	10	−8.75	15
			After ET	10	−5	15
	DyS	Before INT	Before ET	0	−10	10
			After ET	10	0	18.75
		After INT	Before ET	2.5	−8.75	15
			After ET	12.5	0	20
*Ely’s Test*	StS	Before INT	Before ET	130	100	140
			After ET	130	85	140
		After INT	Before ET	132.5	100	140
			After ET	132.5	90	140
	DyS	Before INT	Before ET	130	70	140
			After ET	140	90	140
		After INT	Before ET	127.5	112.5	140
			After ET	140	126.5	140
*Internal*	StS	Before INT	Before ET	50	40	60
*rotation*			After ET	50	40	60
		After INT	Before ET	50	40	60
			After ET	50	40	60
	DyS	Before INT	Before ET	50	45	65
			After ET	60	50	68.75
		After INT	Before ET	50	45	60
			After ET	57.5	50	65
*External*	StS	Before INT	Before ET	60	45	70
*rotation*			After ET	60	50	70
		After INT	Before ET	70	60	70
			After ET	70	60	70
	DyS	Before INT	Before ET	55	40	65
			After ET	60	56.25	70
		After INT	Before ET	60	40	70
			After ET	70	60	75

**Notes.**

Data are presented as median and quartiles. Analysed with a Friedman 2 × 2 repeated measurement ANOVA was used.

## Results

Four children did not complete the intervention because of illness, surgery or pain (Table 2). None of the dropout reasons were related to the exercise intervention. Dynamic standing was performed on statistically higher number of occasions during the exercise intervention period than StS (Table 3). But the total time did not differ between DyS and StS. There was a higher number of non-exercising days during StS than DyS. The wash-out period between the two exercise interventions was 50.3 (24.5) days for all participants (Minimum 14 days; maximum 96 days). For the group starting with StS the wash-out period was 48.5 (26.7) days, (Minimum 18 days; maximum 96 days) and for the group staring with DyS 52.7 (22.8) days, (Minimum 14 days; maximum 76 days). No statistical difference (*p* = 0.71) was found between the two groups, tested with an un-paired students *t*-test.

Statistically significant differences were seen in PROM of the hip before and after exercise testing of StS and DyS analysed with a Friedman test (*p* = 0.001). Further analysis with paired Wilcoxon Signed Ranks Tests revealed statistical differences between before and after exercise test 1 of StS in abduction (*p* = 0.033), external rotation (*p* = 0.002), extension (*p* = 0.029) and before and after exercise test 2 in extension (*p* = 0.016) (Table 4). On the other hand, PROM was found to increase after exercise testing of DyS in all directions at both exercise tests (*p* < 0.001) (Table 4).

When comparing PROM before exercise testing at baseline and after the exercise intervention, statistically significant differences were found between abduction (*p* = 0.001), Ely’s test (*p* = 0.020) and external rotation (*p* = 0.042) at StS and Elys test (*p* = 0.031) and external rotation (*p* = 0.038) at DyS (Table 4).

Changes in PROM before and after exercise tests were statistically significant larger during DyS compared to StS in all movement directions (Table 5).

**Table 5:** The differences in passive range of motion (PROM) in the lower hip between before and after the exercise tests starting and ending the exercise intervention periods of Static (StS) and Dynamic (DyS).

Measure	Exercise test	Exercise intervention	*P*-value	Median	Quartile	
					25th	75th
*Abduction*	Test 1	StS		0	0	5
		Dys	<0.001	5	0	10
	Test 2	StS		0	0	0
		DyS	<0.001	10	5	10
						
*Flexion*	Test 1	StS		0	0	0
		Dys	<0.001	5	5	10
	Test 2	StS		0	0	0
		DyS	<0.001	5	0	10
						
*Extension*	Test 1	StS		0	0	0
		Dys	<0.001	10	5	10
	Test 2	StS		0	0	5
		DyS	<0.001	5	5	10
						
*Ely’s test*	Test 1	StS		0	0	0
		Dys	0.001	5	0	10
	Test 2	StS		0	0	0
		DyS	<0.001	5	0	15
						
*Internal rotation*	Test 1	StS		0	0	5
		Dys	0.019	2.5	0	5
	Test 2	StS		0	0	0
		DyS	<0.001	5	0	10
						
*External rotation*	Test 1	StS		0	0	5
		Dys	0.003	5	5	10
	Test 2	StS		0	0	0
		DyS	<0.001	10	0	10
						


**Notes.**

Data are presented as median and quartiles. Pairwise Wilcoxon Signed Ranks Test was used. A *p*-value less than 0.05 was considered statistically significant.

**Figure 4:**
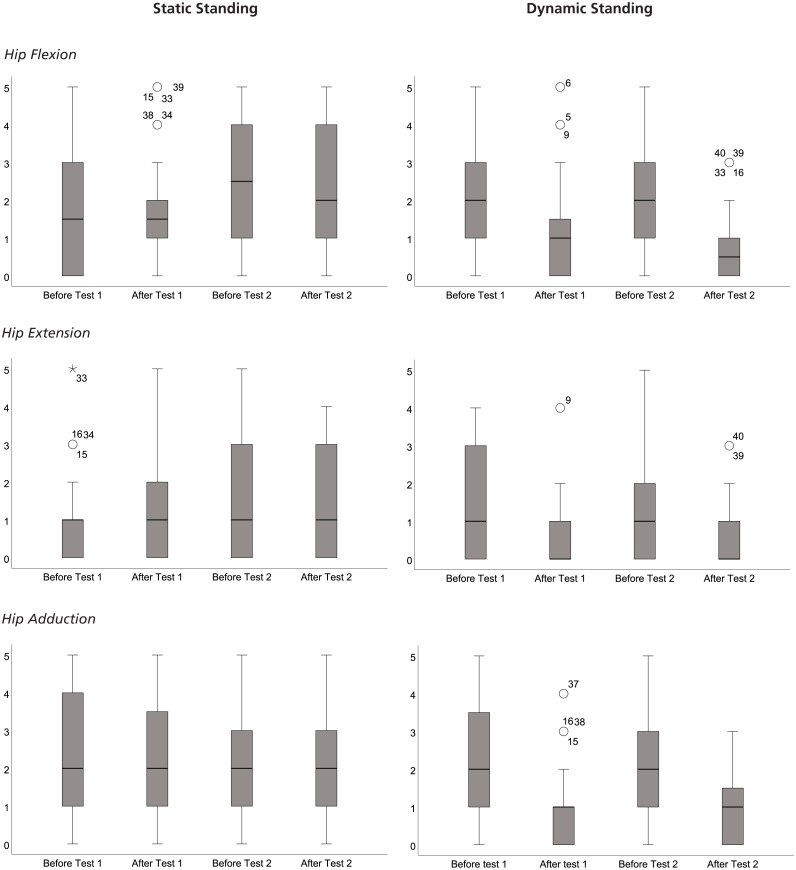
Boxplots of the rating of spaticity, according to Modified Ashworth Scale (Bohannon & Smith, 1987). Shown in the figure are boxplots of the rating of spasticity, according to Modified Ashworth Scale (Bohannon & Smith, 1987), in Hip Flexors (A) Static standing and (B) Dynamic standing), Hip Extensors (C) Static staning and (D) Dynamic standing), and Hip Adductors (E) Static staning and (F) Dynamic standing), before and after the exercise tests starting and ending the exercise intevention periods of Static Standing and Dynamic Standing.

Statistically significant differences were seen in spasticity in muscles around the hip before and after exercise testing of StS and DyS analysed with a Friedman test (Fig. 4). Further analysis with paired Wilcoxon Signed Ranks Tests revealed spasticity to be significantly lower after DyS test 1 (Flexion Before: 2[1:3] vs Flexion After: 1[0:1.75], *p* < 0.001; Extension Before: 1[0:3] v. Hip Extension After: 0 [0:1], *p* < 0.001; Hip Adduction Before: 2[1:3.75] vs Adduction After: 1[0:1], *p* < 0.001) and after Dys test 2 (Flexion Before: 2[1:3] vs. Flexion After: 0.5[0:1], *p* < 0.001; Extension Before: 1[0:2] vs Extension After: 0[0:1] *p* < 0.001; Adduction Before 2[1:3] vs Adduction After: 1[0:1.75], *p* < 0.001) (Fig. 4). After 30 min of StS, the only statistically significant difference was found in Hip flexion (Flexion Before: 1.5[0:3] vs Flexion After: 1.5[1:2], *p* < 0.040) after test 1. Thirty minutes of DyS lowered the spasticity in the muscles around the hip to a statistically significantly higher degree than 30 min of StS (Flexion Test 1 *p* = 0.003; Flexion Test 2 *p* < 0.001; Extension Test 1 *p* < 0.001; Extension Test 2 *p* = 0.001; Adduction Test 1 *p* < 0.001; Adduction Test 2 *p* < 0.001) (Fig. 4). No statistically significant differences in spasticity were found after either DyS or StS, or the four months of exercise training.

## Discussion

We have demonstrated an increased PROM and decreased spasticity in the hip after DyS, whereas almost no statistically significant differences were observed in PROM or spasticity after the Swedish standard care, StS. Dynamic standing was performed at a higher frequency than StS, but the total exercise time during the four months did not differ between DyS and StS on a group level, giving the same dose of exercise, and the two exercise periods were comparable to each other. We decided to have a minimum of two weeks wash-out period since it earlier has been reported that VO _2peak_ decline significantly among sedentary individuals (Neufer, 1989). Others have reported three weeks to be a valid break point, but these studies have been among elite athletes (Mujika & Padilla, 2000; Coyle et al., 1984). Since fitness level influences the detraining effect (Hyatt et al., 2019), we decided to have two weeks as a minimum.

Spasticity may imply a compensation for muscle weakness for some children with CP. However, spasticity may also contribute to muscle shortening, torsional deformities, hip dislocation and/or scoliosis, which often causes pain and can affect motor control, function and activity (Sheean, 2002; Franzen, Hagglund & Alriksson-Schmidt, 2017). Treatment with Botulinum toxin A in order to reduce spasticity has been used since 1993 (Franzen, Hagglund & Alriksson-Schmidt, 2017). StS is also recommended to reduce spasticity and to increase PROM (Paleg, Smith & Glickman, 2013). When planning this study, we chose, inspired from the experiences of our pilot study (Lauruschkus & Tornberg, 2018), to make all assessments in the children’s habitual environment, to ensure a calm, known and secure environment for the testing, and to enable an increased involvement of the children.

Assessments of PROM with a handheld goniometer have been shown to be reliable for abduction (±2.5°) and internal rotation (±2.4°) with errors less than 5 degrees described as standard errors of the measurement (SEM) (McDowell et al., 2000). Ashton, Pickles & Roll (1978) demonstrated an absolute reliability of goniometer assessment of PROM in the hip made by trained physiotherapists among children with CP to vary between ±2.1–4.2° (abduction ±2.5–2.8°; extension ±2.1–3.5°; externa rotation ± 3.5–4.2°) described as SEM. SEM reported in these studies support that the differences found in our study were lager than the expected measurement error (Table 5). After StS no changes in PROM were found (Table 5). On the other hand, PROM increased after DyS exercise testing in median between 5–10° in all motions except for internal rotation at Test 1 where an increase of 2.5° was found (Table 5), meaning that our findings were larger that SEM earlier reported (McDowell et al., 2000; Ashton, Pickles & Roll, 1978) and thereby not explained by random error. This was also seen when comparing StS with DyS, where the differences varied between 2.5−10° (Table 5) which is larger than SEM (McDowell et al., 2000; Ashton, Pickles & Roll, 1978).

Differences in PROM were seen between before and after exercise testing in both StS and DyS. For StS, an increase in PROM was seen in abduction, external rotation, extension after exercise test 1, and in extension after exercise test 2. For DyS, an increase was seen in all movement directions after both test 1 and test 2. When comparing before and after each exercise training period, increases in PROM were found in abduction, extension and external rotation after StS and extension and external rotation after DyS. Changes in PROM were statistically larger after DyS compared to StS. No adverse events were seen, which is an important finding since clinicians have expressed worries that DyS would decrease PROM. Earlier studies have shown a decrease in PROM with age and GMFCS-E&R level (Nordmark et al., 2009), which makes these current findings of clinical interest.

More objective methods to assess spasticity have been suggested (Bar-On et al., 2014b), but we chose to use the Modified Ashworth Scale (Bohannon & Smith, 1987), since this is widely used clinically and within the National Quality Registry CPUP (http://www.cpup.se). It has been shown that spasticity in children with CP increases up to the age of 5 years, and then decreases over time (Nordmark et al., 2009), and that PROM decreases from 2–14 years of age (Nordmark et al., 2009). This information is important to take into consideration for our study. When PROM is expected to decrease, even small changes may have an impact for the children. Since spasticity can have an impact on PROM an interesting finding in our study was that spasticity decreased in all motion directions after both test sessions of DyS but only in flexion after test 1 of StS. DyS lowered spasticity to a statistically higher degree after each test session compared with StS. No differences in spasticity were found after four months of either DyS or StS exercise training. The effects on spasticity seems to be acute, but not adaptable to the exercise. These findings were somewhat surprising, since spasticity has been reported to be velocity dependent (Falisse et al., 2018). In earlier studies, only a few repetitions of the movement have been performed (Bar-On et al., 2014a). In our study, the movements were repeated for 30 min. One can speculate that the decrease in spasticity in our study is a reflection of fatigue in the systems stimulated by the repeated movements during DyS (Allen, Lamb & Westerblad, 2008). This speculation needs to be tested in future studies.

Limitations of our study were the difficulties of standardising the exercise interventions. The length of each StS session varied because the children use the standard care differently due to individual reasons. Likewise, it was hard to standardise walking speed and exercise time during DyS due to the individual child’s preferences. After analysing the exercise diaries, we saw that the children statistically got the same dose of exercise both during StS and DyS, which made us decide that a comparison between the two interventions was possible. Additionally, the daily delivery of the intervention was performed by parents, school personnel and personal assistants of the children, which might have introduced variation in the delivery. All, parents, personnel and assistants got the same information and education to minimise this. The follow up half time during DyS, also supported the delivery of the intervention. The wide ranges of days in the wash-out period could also be a problem. In the analysis of the number of days within the wash-out period we couldn’t find any statistical differences between the children starting with StS intervention and those starting with the DyS intervention, which made us decide that this most likely didn’t influence our results on a group level. We also found that no child had a shorter period than 14 days, which was our criteria for the wash-out. Indeed, the average child had a wash-out of 50 day, well within the limits that have been suggested for stabilisation on a new activity level (Coyle et al., 1984). Another limitation of our study is that it wasn’t possible to blind the participants to their intervention and to the assessors. Our results have to be interpreted in light of this.

### Implications

None of the standing modalities seemed to decrease PROM or increase spasticity on a group level, over time. This is to our knowledge the first structured study on the effects of DyS on PROM and spasticity in the hip, which is why it is important to point out that this type of standing does not cause harm. Since, for non-ambulatory children with CP, DyS at present only can be performed in an Innowalk, DyS may be introduced as a good complement to the standard care. Disadvantages of the use of an Innowalk at home, are that it is expensive to purchase and that it therefore may not be offered by different health care systems. Additionally, it can only be adjusted by specially trained personal.

## Conclusions

Thirty minutes of dynamic standing increased PROM and decreased spasticity, and four months of DyS increased PROM but did not decrease spasticity among non-ambulatory children with CP on a group level. These results can help develop individualised standing recommendations.

##  Supplemental Information

10.7717/peerj.8561/supp-1 Supplemental Information 1 Raw data of PROM

10.7717/peerj.8561/supp-2 Supplemental Information 2 Raw data of spasticity assessments

10.7717/peerj.8561/supp-3 Supplemental Information 3 The CONSORT Checklist for the study

10.7717/peerj.8561/supp-4 Supplemental Information 4 The study protocol in ISRCTN

## Additional Information and Declarations

### Data Availability

The following information was supplied regarding data availability: The raw measurements are available in the Supplementary Files.

### Clinical Trial Registration

The following information was supplied regarding Clinical Trial registration:
ISRCTN10569363.
