# Supplementary material for: Non-ambulatory children with cerebral palsy: effects of four months of static and dynamic standing exercise on passive range of motion and spasticity in the hip
Source: PeerJ. 2020 Mar 17;8:e8561. doi: 10.7717/peerj.8561 (PMC7083156; doi:10.7717/peerj.8561)
Supplement: Supplemental Information 4 [file peerj-08-8561-s004.pdf]

ISRCTN registry

ISRCTN10569363 https://doi.org/10.1186/ISRCTN10569363 [https://doi.org/10.1186/ISRCTN10569363]

Comparing static standing and dynamic supported standing in children and young people with cerebral palsy who are not able to walk independently

CrossMark [#]

Condition category
Nervous System Diseases
Date applied
06/12/2018
Date assigned
07/12/2018
Last edited
07/12/2018

Prospective/Retrospective
Retrospectively registered
Overall trial status
Completed
Recruitment status
No longer recruiting

Plain English Summary

Background and study aims
Non-ambulatory (not able to walk) children and young people with cerebral palsy (CP) are not physically active and have a lot of sedentary time which has significant health risks. The standard care for those children in Sweden includes static (non-mobile) standing training (StS) in standing frames for 45-90 minutes daily. This helps with bone strength, breathing and digestive function. Innowalk is a frame that can hold a person upright and support their weight while moving their legs as if walking. Parents of non-ambulatory children with CP performing dynamic standing (DyS) in an upright weight-bearing position with the motorised medical device Innowalk report effects not seen during StS. In a pilot study with 7 children the responses of one bout of 30 minutes StS and 30 minutes DyS were compared. The results from the pilot study showed that the study design was feasible and that there were differences in the physical responses to the two standing regimes. This study aims to investigate to see whether there are differences in the response to 4 months of StS and DyS in metabolic response, breathing, pain, gastrointestinal function, quality of life, physical activity, temperature in the feet, spasticity (muscle stiffness) and Passive Range of Motion in the hands and feet among non-ambulatory children and young people with CP.

Who can participate?
Non-ambulatory children and young people with CP aged 2-17, living in the Skane Region, Sweden

What does the study involve?
Each child performs 4 months of static standing and 4 months of dynamic standing in a home-setting, including a wash-out period of 2 weeks between the exercise periods. Assessments and measurements are made at 4 occasions, at the beginning and end of each exercise period.

What are the possible benefits and risks of participating?
Benefits include the knowledge gained about different types of standing. The study assessments and measurements were performed in a home setting to make the children/young people more comfortable and to minimise the possible inconveniences from the measurements. The parents could end their participation in the study at any time.

Where is the study run from?
Lund University (Sweden)

When is study starting and how long is it expected to run for?
September 2016 to May 2018

Who is funding the study?
1. Stiftelsen för Rörelsehindrade i Skane, Sweden (Foundation for people with physical disabilities in the Skane Region, Sweden)
2. The Promobilia foundation (Sweden)
3. Swedish National Association for Disabled Children and Young People (Sweden)
4. Linnéa och Josef Carlssons stiftelse, Sweden (Linnéa and Josef Carlssons Foundation, Sweden)

Who is the main contact?
Dr Katarina Lauruschkus
katarina.lauruschkus@med.lu.se

Trial website

N/A [N/A]

Contact information

Type

Scientific

Primary contact

Dr Katarina Lauruschkus

ORCID ID

http://orcid.org/0000-0002-1801-8503 [http://orcid.org/0000-0002-1801-8503]

Contact details

Box 157
Lund
22100

Sweden
+46 707 51 98 88
katarina.lauruschkus@med.lu.se [mailto:katarina.lauruschkus@med.lu.se]

Additional identifiers

EudraCT number

ClinicalTrials.gov number

Protocol/serial number

N/A

Study information

Scientific title

Effects of 4 months static standing compared to 4 months dynamic supported standing among non-ambulatory children and young people with cerebral palsy

Acronym

Study hypothesis

The study hypotheses are that there are differences in the response to 4 months exercise of Static Standing and Dynamic Standing in metabolic response, respiration, physical activity, temperature at the feet, quality of life, pain, gastrointestinal function, spasticity and Passive Range Of Motion (PROM) in the hips, knee and ankle joints among non-ambulatory children and young people with cerebral palsy.

Ethics approval

Regional Ethical Review Board at Lund University, Sweden, 30/03/2017, ref: LU-Dnr 2017/67

Study design

Within-patient controlled trial

Primary study design

Interventional

Secondary study design

Non randomised study

Trial setting

Home

Trial type

Treatment

Patient information sheet

Not available in web format, please use contact details to request a participant information sheet

Condition

Cerebral palsy

Intervention

The standard care in Sweden for non-ambulatory children and young people with cerebral palsy (CP) includes daily static supported standing. The standing exercise training in standing frames is a static standing (StS) exercise where the child is fixated in the standing frame. No movements in the lower body can be achieved but making standing in an upright position possible. The motorised medical device Innowalk gives an opportunity to experience walking movements in an upright weight-bearing position, making dynamic standing (DyS) possible. The participants performed 4 months of standing for 30-60 minutes/day in StS and DyS at their home or preschool/school. Measurements were performed and questionnaires filled in at four occasions, at the beginning and end of each exercise period. Assessments of spasticity and passive range of motion (PROM) were performed and thereafter, capillary blood sample was taken and heart rate belt were put on. The child was positioned in either the standing frame or in the Innowalk. If the Innowalk was to be used it was individually adjusted to the child. When the child was in an upright position the airtight mask covering mouth and nose was put on. The indirect caloric assessment in a standing position was performed for 30 minutes. Temperature of the feet was measured every 10th minute during standing. After 30 minutes of standing the child was lifted down on a mat and a capillary blood sample was taken and assessments of spasticity and PROM were performed once more.

Intervention type

Device

Phase

Drug names

Primary outcome measure

Respiratory gas exchange VO2, VCO2, VE and BF using with an airtight mask covering the mouth and nose while standing for 30 minutes at all four test points (at baseline and after 4 months of StS or DyS)

Secondary outcome measures

1. Metabolic response: capillary blood samples were taken from a fingertip for blood glucose and blood lactate analysis before and after standing for 30 minutes in StS or DyS at all four test points (at baseline and after 4 months of StS or DyS)
2. Heart rate monitored with a Polar belt while standing for 30 minutes in StS or DyS at all four test points (at baseline and after 4 months of StS or DyS) at all four test points (at baseline and after 4 months of StS or DyS)
3. Spasticity in hip flexors and extenders, adductors, hamstrings, knee extenders and gastrocnemius assessed by the Modified Ashworth Scale before and after standing for 30 minutes in StS or DyS at

all four test points (at baseline and after 4 months of StS or DyS)
4. Passive Range Of Motion (PROM) in the hips, knee and ankle joints measured by goniometry before and after standing for 30 minutes in StS or DyS at all four test points (at baseline and after 4 months of StS or DyS)
5. Pain was assessed by questionnaire before, during and after standing for 30 minutes in StS or DyS at all four test points (at baseline and after 4 months of StS or DyS)
6. Body temperature assessed using infrared thermometer at both feet at rest before standing, 1 minute before the test started, at 10, 20 and 30 minutes of standing and at rest after standing for 30 minutes in StS or DyS at all four test points (at baseline and after 4 months of StS or DyS)
7. Gastro-intestinal function questionnaires was completed at all four test points (at baseline and after 4 four months of StS respectively DyS).
8. Quality of life was assessed by the CPChild assessment at all four test points (at baseline and after 4 four months of StS respectively DyS).

Overall trial start date

01/09/2016

Overall trial end date

17/05/2018

Reason abandoned (if study stopped)

Eligibility

Participant inclusion criteria

1. Cerebral palsy, with Gross Motor Function Classification System (GMFCS) level IV and V (non-ambulatory)
2. Aged 2-17 years
3. Living in the Skane Region in Southern Sweden
4. Performing static standing as standard care
5. Body length 80-190 cm

Participant type

Patient

Age group

Child

Gender

Both

Target number of participants

24

Participant exclusion criteria

1. Body length less than 80 cm or more than 190 cm
2. Body weight more than 90 kg, due to the restrictions of the device producers
3. Planned orthopaedic surgery in the spine and lower extremities

Recruitment start date

01/04/2017

Recruitment end date

01/06/2017

Locations

Countries of recruitment

Sweden

Trial participating centre

Health Sciences Center, Lund University
Box 157
Lund
22100
Sweden

Sponsor information

Organisation

Lund University, Faculty of Medicine

Sponsor details

Box 117
Lund
22100
Sweden
+46 (0)46 222 00 00
info@med.lu.se [mailto:info@med.lu.se]

Sponsor type

University/education

Website

https://www.med.lu.se/ [https://www.med.lu.se/]

Funders

Funder type

Other

Funder name

Stiftelsen för Rörelsehindrade i Skane, Sweden (Foundation for people with physical disabilities in the Skane Region, Sweden)

Alternative name(s)

Funding Body Type

Funding Body Subtype

Location

Results and Publications

Publication and dissemination plan

The study protocol will be available on request. 3-6 scientific papers and presentations at conferences are planned.

IPD sharing statement
Participant level data will be available on request from Dr Katarina Lauruschkus (katarina.lauruschkus@med.lu.se) from December 2018 to November 2028. Consent from the participants' parents as their legal guardians was obtained. All data presented are anonymised, and there is a code list locked in at Lund University.

Intention to publish date

30/09/2019

Participant level data

Available on request

Basic results (scientific)

Publication list

Publication citations

Additional files

Editorial Notes
